# Sexual dysfunction in chronic hepatitis B: a comprehensive review of pathophysiology, clinical burden, and integrated management

**DOI:** 10.3389/fpubh.2026.1865911

**Published:** 2026-08-04

**Authors:** Sahar Muzammal, Cheng-Run Song, Jiang-Nan Peng, Yong-Hong Wang, En-Qiang Chen

**Affiliations:** Center of Infectious Diseases, West China Hospital, Sichuan University, Chengdu, China

**Keywords:** antiviral therapy, biopsychosocial model, chronic hepatitis B, integrated management, sexual dysfunction

## Abstract

Chronic hepatitis B (CHB) affects approximately 240 million people worldwide. Advances in antiviral therapy have shifted CHB management toward long-term, multidimensional care, in which sexual health is closely linked to psychological wellbeing, intimate relationships, and patient-clinician communication. However, sexual dysfunction (SD) in CHB remains insufficiently characterized and is not yet routinely integrated into clinical management. This narrative review synthesizes evidence focused on CHB regarding the epidemiology, clinical burden, pathophysiological mechanisms, and antiviral treatment implications of SD across disease stages and treatment contexts. Reported prevalence estimates vary widely, ranging from 8.6 to 63.1% across heterogeneous HBV-related studies. Mechanistically, SD in CHB appears to reflect interactions among biological vulnerability, psychological stressors, and lifestyle determinants. Evidence on treatment effects remains limited, but available data do not indicate a consistent adverse effect of nucleos(t)ide analogues on sexual function, whereas interferon-based regimens may indirectly contribute to SD through fatigue and neuropsychiatric symptoms. Accordingly, we propose an integrated management framework comprising proactive screening, multidimensional assessment, individualized intervention, and longitudinal monitoring. In practice, this framework emphasizes validated sexual function instruments, concurrent assessment of psychological distress, transmission concerns, closer monitoring of patients at higher risk, targeted symptom management, and coordinated multidisciplinary support when needed. Future research should prioritize prospective longitudinal studies, sex-specific analyses, standardized sexual function instruments, and inclusion of sexual health as a patient-reported endpoint in antiviral therapy trials to better define the burden, mechanisms, and management of SD in CHB.

## 1 Introduction

Chronic hepatitis B (CHB) poses a continuing threat to global public health, with an estimated 240 million individuals infected and approximately 1.1 million deaths in 2024 resulting from complications of hepatitis B virus (HBV) infection, such as cirrhosis and hepatocellular carcinoma (HCC) (1). The clinical and economic burden of CHB is profound, contributing to substantial healthcare costs and loss of workforce productivity, a trend further exacerbated by the demographic shift toward an older HBV-infected population (2). Although the management of CHB has long focused on achieving virological suppression to halt liver disease progression, it is increasingly recognized that CHB exerts effects far beyond the liver, substantially compromising patient’s quality of life and functional status (3). Sexual dysfunction (SD) is a common but frequently overlooked extrahepatic manifestation of CHB (4). It refers to clinically significant disturbances in the sexual response cycle, which include impairments in desire, arousal, orgasm, or pain, and are accompanied by personal distress (5). SD in CHB constitutes a core component of the disease’s multisystem impact rather than an incidental finding. Its impact may extend beyond sexual intimacy and overlap with broader patient-reported burdens in CHB, including psychological distress, impaired quality of life, self-management difficulties, and concerns within intimate relationships (6–8). Despite its high prevalence and clinical significance, SD is often overlooked in routine hepatology practice owing to time constraints, lack of clinician training, and the topic’s perceived sensitivity, resulting in a critical gap in comprehensive patient care.

Given the complex and multifactorial pathophysiology of SD in CHB, the biopsychosocial framework is indispensable for a comprehensive understanding, moving beyond the limitations of a purely biomedical perspective (9). This model conceptualizes SD as resulting from the intertwined effects of direct biological insults and potent psychosocial stressors. Key biological contributors include liver dysfunction-induced hormonal dysregulation, chronic inflammation-mediated endothelial and neurological impairment, and contributions from comorbidities such as metabolic syndrome. Concurrently, significant psychosocial factors encompass disease-related anxiety, depression, and the pervasive social stigma associated with HBV (10). These psychosocial stressors can, in turn, exacerbate underlying biological dysfunction and sustain a self-perpetuating cycle of distress, social isolation, and relationship strain (11).

Previous reviews and meta-analyses have advanced understanding of sexual dysfunction in chronic liver disease. However, these works largely focused on broader chronic liver disease populations rather than CHB-specific clinical contexts. The present review builds on this literature by providing a CHB-centered synthesis of available evidence across different disease stages, from non-cirrhotic to cirrhotic CHB. Specifically, it summarizes prevalence estimates and sex-specific manifestations of SD in CHB patients; evaluates the potential impact of antiviral treatment and highlights the need to incorporate sexual health patient-reported outcome measures in future research; and outlines a practical framework for screening, assessment, and management. This review aims to improve recognition of SD as part of comprehensive CHB care and support practical, patient-centered assessment and management.

This article is a narrative review that synthesizes heterogeneous evidence relevant to SD in CHB. We searched PubMed, Embase, and Web of Science from inception to March 2026 using combinations of the following terms: ‘hepatitis B chronic’, ‘sexual dysfunction’, ‘erectile dysfunction’, ‘libido’, ‘antiviral agents’, ‘interferon’, ‘nucleos(t)ide analogues’, ‘biopsychosocial’, and ‘quality of life’. Relevant original studies, reviews, and clinical guidelines were considered to support the narrative synthesis. We prioritized CHB-specific primary studies for epidemiological and clinical statements, while evidence from other chronic diseases (cirrhosis, NAFLD, diabetes) was used only to supplement mechanistic understanding, with explicit labeling as indirect evidence.

## 2 Epidemiology and clinical significance of sexual dysfunction in CHB

Available evidence suggests that SD are underrecognized concerns in CHB (7, 12). However, the current literature is heterogeneous because of differences in assessment tools, disease severity, cirrhosis status, treatment background, sex distribution, and study population. This well-established burden positions SD as a major determinant of health-related quality of life and a significant clinical challenge in CHB (13).

Reported estimates of SD in CHB vary substantially, ranging from 8.6 to 47.8% across available studies (12, 14). In a larger CHB cohort, the overall prevalence of SD was 25.4%, with higher rates among patients with poorer liver function reserve, greater fibrosis burden, and more severe depression (7). These findings indicate that SD represents a clinically relevant but heterogeneous sexual health burden in CHB and HBV-related liver disease. The clinical manifestations of SD differ meaningfully by gender, reflecting distinct pathophysiological and psychosocial underpinnings (5). Among male patients, erectile dysfunction (ED) is the most commonly reported and investigated condition. Other domains of male sexual function, including sexual desire and ejaculatory function, may also be affected, but their CHB-specific prevalence remains insufficiently characterized (15). The high prevalence of ED in this population underscores the prominent role of underlying vascular and hormonal disturbances in the pathophysiology of CHB-related SD.

Female SD has also been reported in CHB populations, but remains less well characterized than male ED. Therefore, we summarized available data from mixed chronic liver disease cohorts, in which reported female SD prevalence ranged from 13.6 to 63.1% (7, 12, 16). Reported symptoms include reduced desire, arousal or lubrication difficulties, dyspareunia, and orgasmic difficulties (7). These manifestations are often overlooked in hepatology settings, despite their substantial negative effects on personal wellbeing, body image, and intimate relationships (17, 18). Importantly, the evidence for female sexual dysfunction in CHB is considerably scarcer than for male erectile dysfunction; most female-specific data derive from small cross-sectional studies, and larger prospective cohorts are urgently needed.

Notably, SD has relevance to CHB management beyond sexual wellbeing. Previous studies have associated SD in CHB with depressive symptoms and impaired quality of life, while HBV-related stigma and psychological distress have been linked to poorer self-management and difficulties in intimate relationships (8, 12, 19). Although direct evidence that SD itself reduces antiviral adherence remains limited, these overlapping burdens may influence symptom interpretation, treatment-related communication, and engagement with long-term care. Systematic assessment of SD may therefore help identify unmet psychological, relational, and self-management needs that are not captured by routine virological or biochemical monitoring.

Appropriate assessment of SD is essential for its recognition and management in patients with CHB. Although several validated instruments are available, including the International Index of Erectile Function (IIEF) for men and the Female Sexual Function Index (FSFI) for women, standardized sexual health assessment remains insufficiently integrated into routine CHB care (20–22). This lack of systematic screening not only perpetuates the under-recognition of SD but also constitutes a missed opportunity to provide comprehensive patient care. Incorporating these well-validated questionnaires into annual or biannual clinical evaluations could substantially enhance detection rates and enable timely intervention, thereby helping to disrupt the cycle of distress and treatment non-adherence.

As shown in Table 1, the epidemiological and clinical profile of SD in CHB highlights its high prevalence, gender-specific manifestations (e.g., ED in men, arousal difficulties in women), and critical impact on treatment adherence and clinical outcomes. This table underscores the need for routine screening to address this underdiagnosed comorbidity.

**Table 1:** Epidemiological and clinical profile of sexual dysfunction in chronic Hepatitis B.

Aspect	Overall population	Male patients	Female patients	Clinical implications
Prevalence of SD	8.6–63.1%^1^	8.6–47.8%^1^	13.6–63.1%^2^	SD is a highly prevalent, yet under-diagnosed, comorbidity that warrants routine screening.
Common manifestations	N/A	Erectile dysfunction (ED), reduced libido, ejaculatory disorders.	Diminished desire, arousal difficulties, dyspareunia, anorgasmia.	Recognition of gender-specific presentations is essential for targeted history-taking and management (5).
Impact on treatment adherence	Associated with reduced adherence to antiviral therapy.	Often linked to depression and body image concerns.	Frequently associated with stigma and fertility anxieties.	SD directly compromises long-term treatment success and should be addressed as part of adherence counseling (19).
Current assessment practices	Underutilization of validated screening tools.	IIEF rarely used in clinical practice.	FSFI infrequently administered.	Lack of standardized assessment is a major barrier to recognition and intervention.

1 Reported estimates varied substantially across studies because of differences in disease severity, cirrhosis status, assessment tools, treatment background, and study populations.

2 Female estimates were summarized mainly from mixed chronic liver disease or mixed CHB/CHC cohorts because female CHB-specific studies are limited.

## 3 Pathophysiology: a biopsychosocial framework

**Figure 1:**
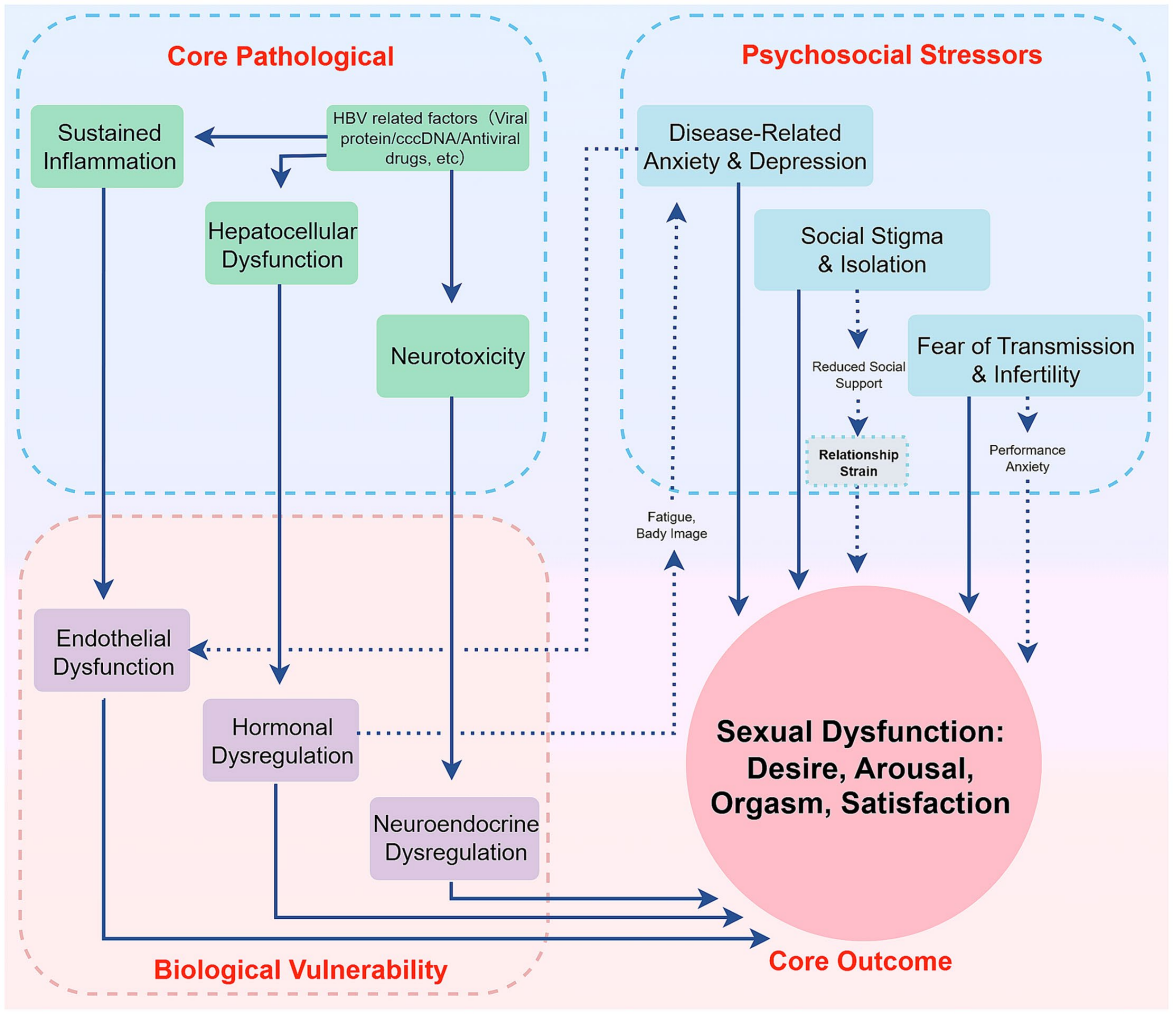
Multifactorial pathophysiology of sexual dysfunction in chronic Hepatitis B: a biopsychosocial model. This schematic illustrates the integrated pathophysiological model of sexual dysfunction (SD) in chronic Hepatitis B (CHB). The core pathological processes triggered by chronic HBV infection-sustained inflammation, hepatocellular dysfunction, and neurotoxicity-converge to create a state of biological vulnerability (left side). These biological pathways interact synergistically and bidirectionally with a significant burden of psychosocial stressors (right side), which are often exacerbated by the disease and its treatment. This dynamic interplay between biological, psychological, and social domains directly drives the high prevalence and complexity of SD in CHB, positioning it as a central determinant of patient-reported outcomes.

Sexual dysfunction in CHB does not arise from a single cause but results from the complex interplay of biological, psychological, and social factors (Figure 1). Notably, the strength and applicability of these mechanisms vary across disease stages. Evidence for biological mechanisms, such as altered sex hormone metabolism, is derived mainly from advanced CHB or cirrhosis. Yet psychosocial mechanisms, including depressive symptoms, anxiety, stigma, and intimate relationship stress, may offer a more applicable explanation for SD in clinically stable CHB populations. Figure 1 presents the multifactorial pathophysiological model of SD in CHB, illustrating how biological vulnerabilities (e.g., hormonal dysregulation, inflammation, neuropathy) interact with psychosocial stressors (e.g., anxiety, stigma) to drive the high prevalence and complexity of this condition.

### 3.1 Biological mechanisms

Among biological factors, existing evidence most consistently links SD to overall liver disease burden, including fibrosis progression, impaired liver function reserve, and cirrhosis (7, 23, 24). This involves compromised synthesis of sex hormone-binding globulin (SHBG) and defective inactivation of steroid hormones (7, 24, 25). In male patients, a central manifestation is hypogonadism, driven by a combination of diminished SHBG production and reduced estrogen clearance, which promotes a relative hyperestrogenic environment. This altered hormonal milieu suppresses the hypothalamic–pituitary-gonadal (HPG) axis, consequently inhibiting testosterone synthesis. Low serum testosterone levels, which are closely linked to CHB severity, constitute a major pathogenic factor in sexual dysfunction, notably causing reduced libido and erectile dysfunction (7, 24, 26). In female patients, suppression of the HPG axis and altered estrogen-progesterone balance have been described in chronic liver disease (7, 24). These abnormalities may manifest as menstrual irregularities and may contribute to SD related symptoms, including reduced sexual desire, impaired lubrication, and dyspareunia (27). However, their direct contribution to female SD in CHB remains insufficiently established. Direct evidence for this mechanism in women with CHB is lacking, but analogous processes have been described in other chronic inflammatory conditions. Therefore, this remains a hypothesis requiring dedicated study in female CHB populations. Compounding this issue, malnutrition in advanced-stage CHB further aggravates hormonal deficits by limiting the availability of critical precursors for steroid hormone production (24, 28).

Persistent HBV infection is characterized by chronic inflammation, which elevates circulating levels of pro-inflammatory cytokines such as TNF-*α* and IL-6. These cytokines play a central role in the development of endothelial dysfunction by attenuating the bioavailability of vasodilatory nitric oxide (NO) (22, 29, 30). In the male genitourinary system, this vascular pathology manifests as impaired relaxation of cavernosal smooth muscle, resulting in insufficient penile blood flow and frank erectile dysfunction (22, 29, 31). Objective evidence from Doppler ultrasonography confirms reduced peak systolic velocity and aberrant penile hemodynamics in this patient population. In female patients, vascular responsiveness and NO/eNOS signaling are also involved in genital arousal and lubrication, but its specific role in HBV-related female SD has not been well established (22, 29, 32).

Advanced liver disease will also have adverse effects on the nervous system, which makes patients susceptible to peripheral and autonomic neuropathy (33). The resulting impairment disrupts delicate autonomic circuits essential for erectile function in males and for arousal and orgasmic responses across sexes (34). Concurrently, persistent inflammatory states may disturb neurotransmitter balance, further compromising neural regulation of sexual response. In addition, metabolic and cardiovascular comorbidities are increasingly recognized as clinically relevant factors in CHB populations and may increase susceptibility to SD through vascular dysfunction, hormonal disturbance, and systemic inflammation (31, 35). Together, neural impairment, persistent inflammation, and metabolic or cardiovascular comorbidities may converge with liver-related endocrine and vascular disturbances to impair sexual health.

### 3.2 Psychological and social factors

The psychological burden associated with CHB is considerable and represents an independent risk factor for sexual dysfunction. Anxiety and depression are frequently observed in this population, arising from concerns about diagnosis, disease progression, viral transmission to partners, and fertility prospects (10, 19). The prevalence of depression and anxiety in CHB patients treated with oral antiviral drugs were 33.0 and 38.3%, respectively (6). Standardized psychological assessment may help identify depressive and anxiety symptoms in CHB patients and guide timely referral or intervention. These mood disturbances markedly diminish libido and disrupt the psychological arousal necessary for satisfactory sexual function (11).

Social stigma related to HBV, perpetuated by misconceptions about transmission, contributes to discrimination and internalized shame. This stigma can prompt patients to avoid intimate relationships for fear of rejection or transmitting the virus (8). Within established relationships, the combined challenges of managing CHB and sexual dysfunction often lead to communication breakdowns, interpersonal conflict, and diminished emotional and physical intimacy, further aggravating sexual difficulties (36, 37). Additionally, the psychological impact of antiviral therapy merits consideration; certain agents, particularly interferon, are known to induce depression and fatigue, which in turn contribute to sexual dysfunction (38).

### 3.3 Lifestyle factors

Lifestyle factors represent important modifiable determinants of SD in CHB. Disease-related fatigue and apprehension about worsening their health status frequently result in reduced physical activity among patients. This behavioral shift contributes to diminished cardiovascular fitness, impaired endothelial function, and lowered energy reserves-all of which adversely influence sexual response (7). Concurrently, symptoms such as anorexia, nausea, and metabolic dysregulation may precipitate either nutritional deficits or excessive weight gain. Both malnutrition and obesity disrupt endocrine homeostasis and reduce physiological energy availability, thereby establishing a suboptimal milieu for sexual health (39, 40). Targeted modification of these lifestyle elements offers a valuable avenue for non-pharmacological management in this population. These psychosocial stressors are not merely consequences; they actively exacerbate underlying biological dysfunction, creating a self-perpetuating cycle.

## 4 The impact of antiviral therapy on sexual function

The effect of antiviral therapy on sexual function in CHB represents a critical consideration in clinical management (41). Although successful viral suppression and hepatic improvement would be expected to confer benefits, the specific properties of antiviral agents can exert variable effects. A clear understanding of these differential impacts is essential for optimizing treatment selection and managing patient expectations (42).

### 4.1 Nucleos(t)ide analogues: a generally neutral profile

Nucleos(t)ide analogues (NAs), including entecavir, tenofovir disoproxil fumarate, and tenofovir alafenamide, constitute the first-line treatment for CHB, prized for their potent efficacy and high barrier to resistance (43). Regarding sexual health, available evidence does not support a consistent direct causal relationship between NAs and SD. Hypotheses regarding subtle differences among individual NAs remain inadequately substantiated. Mitochondrial alterations have been reported during NAs therapy in CHB patients, and could theoretically influence steroidogenesis (44). However, the only CHB-specific study directly comparing these agents found no difference in sexual function outcomes (41). Given the lack of robust clinical corroboration, we consider any inter-drug difference speculative. Nevertheless, we acknowledge that this is an open question deserving of larger, adequately powered trials.

When sexual dysfunction arises during NAs therapy, clinicians should prioritize a comprehensive assessment to identify alternative etiologies. Symptoms are more likely attributable to the natural progression of advanced liver disease, which may manifest as worsening hypogonadism, or to the onset or exacerbation of psychological distress, or to the development of other comorbidities such as diabetes mellitus. These factors generally represent a more plausible explanation than a direct pharmacological effect of the NAs. A clinical study reported improved sexual function and semen quality after entecavir treatment in male patients with CHB, but this improvement may reflect better liver function and overall physical condition rather than a specific drug effect on sexual function (45).

### 4.2 Interferon-based regimens: a significant concern

In contrast to NAs, interferon-based regimens, particularly pegylated interferon-alpha (PegIFN-*α*), are associated with a well-documented and significantly higher burden of SD. The adverse effects of interferon on sexual function are primarily indirect, mediated through its characteristic and often profound side-effect profile. Debilitating fatigue, reported in 39% of patients, directly diminishes libido and the physical energy required for sexual activity (46). More significantly, interferon therapy is notorious for inducing neuropsychiatric effects, including major depression, irritability, and emotional lability in 3–42.4% of patients (38, 47, 48). These effects are thought to stem from cytokine-induced alterations in central neurotransmitter pathways, particularly those involving serotonin and dopamine (49). Given that depression is one of the strongest predictors of SD, it acts as a powerful mediator, substantially impairing sexual desire and satisfaction. Although CHB-specific evidence remains limited, this mechanistic rationale is indirectly supported by HCV studies reporting declines in sexual desire, sexual function, and sexual satisfaction during interferon-based therapy (50).

Although the use of interferon has declined in many settings with the availability of superior NAs, its distinct and substantial risk profile for SD remains highly relevant for selected patient populations and in resource-limited settings. This history underscores the importance of proactively assessing sexual health when deploying therapies with significant central nervous system effects.

### 4.3 Implications for future antiviral development

The development of novel antiviral classes, such as capsid assembly modulators and entry inhibitors, makes the systematic assessment of their impact on sexual health more critical than ever. It is strongly recommended that future clinical trials for these agents prospectively integrate validated patient-reported outcome measures (PROMs), such as the IIEF and FSFI, as predefined secondary endpoints. This proactive approach is indispensable for comprehensively characterizing the safety profile of new therapies and ensuring that treatment advancements align with the principles of holistic, patient-centered care. As shown in Table 2, the key pathophysiological pathways of CHB-related SD include hormonal dysregulation (e.g., altered SHBG, estrogen/testosterone imbalance), endothelial dysfunction (e.g., reduced NO bioavailability), neurological impairment (e.g., hyperbilirubinemia-induced neurotoxicity), psychological distress (e.g., anxiety, depression), and lifestyle factors (e.g., malnutrition, obesity). These mechanisms collectively explain the diverse manifestations of SD in CHB patients.

**Table 2:** Key pathophysiological pathways in CHB-related sexual dysfunction.

Mechanism	Pathophysiological pathway	Impact on sexual function
Hormonal dysregulation	Impaired hepatic metabolism → Altered SHBG, ↑estrogen/↓testosterone ratio; malnutrition.	M: Reduced libido, erectile dysfunction. F: Vaginal dryness, dyspareunia, anovulation, reduced desire (7, 24).
Endothelial dysfunction	Chronic inflammation (↑TNF-*α*, IL-6) → ↓NO bioavailability → Impaired vasodilation.	M: Erectile dysfunction due to impaired cavernosal relaxation. F: Impaired genital arousal and lubrication (22, 29).
Neurological impairment	Neurotoxicity from hyperbilirubinemia; autonomic neuropathy; altered neurotransmission.	Diminished arousal, delayed orgasm, erectile difficulties.
Psychological distress	Anxiety, depression, stigma, relationship strain, fear of transmission.	Inhibited desire, psychological arousal, and satisfaction; performance anxiety.
Lifestyle factors	Sedentary behavior, obesity, malnutrition.	Exacerbates endothelial, hormonal, and psychological vulnerabilities (74).

## 5 Clinical management: an integrated, proactive approach

**Figure 2:**
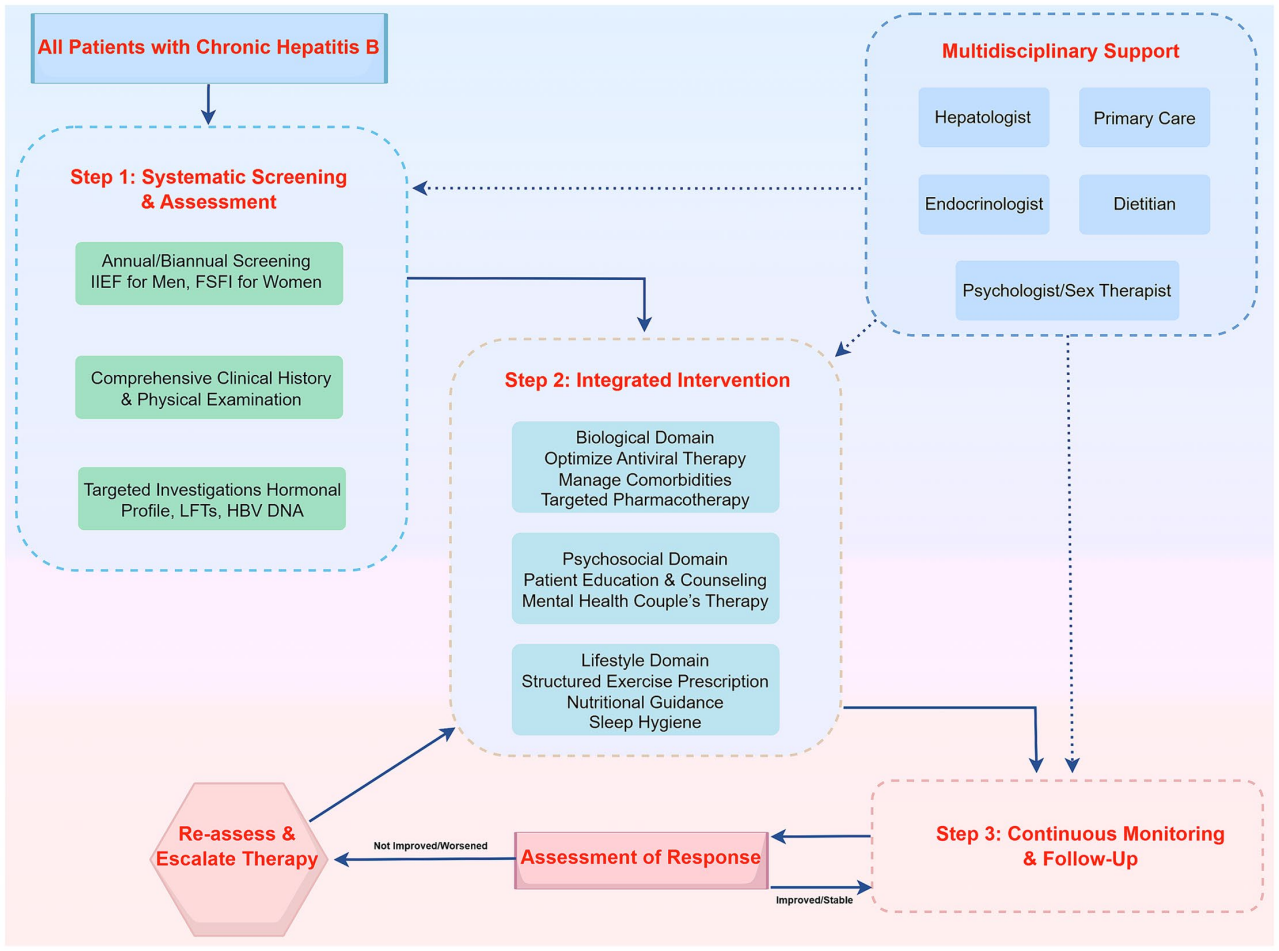
Integrated clinical management pathway for sexual dysfunction in patients with chronic Hepatitis B. This figure proposes a proactive, multidisciplinary clinical pathway for managing sexual dysfunction (SD) in CHB. The algorithm advocates a paradigm shift from reactive care to systematic screening using validated tools (e.g., IIEF, FSFI). Following a comprehensive assessment, an individualized management plan is implemented, addressing biological, psychosocial, and lifestyle factors concurrently and synergistically. The pathway emphasizes continuous monitoring and follow-up to create a closed-loop system, ensuring sustained improvement in sexual health and overall quality of life.

The effective management of sexual dysfunction in patients with CHB necessitates a paradigm shift from reactive, symptom-focused care toward a proactive, integrated, and multidisciplinary strategy informed by the biopsychosocial model. The following framework delineates a systematic clinical pathway that prioritizes early detection and comprehensive intervention. As shown in Figure 2, this integrated clinical management pathway for sexual dysfunction in CHB patients emphasizes a stepwise approach: (1) Routine screening with IIEF/FSFI; (2) Comprehensive assessment of biological, psychological, and lifestyle factors; (3) Targeted interventions (medical, psychological, lifestyle); and (4) Continuous monitoring. This figure visually guides clinicians through the core components of holistic SD management. In the absence of randomized controlled trials for chronic hepatitis B, we point out that recommendations are based on evidence and expert consensus in other chronic disease populations (e.g., diabetes, cardiovascular disease, general psychological medicine).

### 5.1 Step 1: systematic screening and comprehensive assessment

Routine and systematic identification of SD forms the cornerstone of clinical management (51). We recommend implementing annual or biannual screening for all CHB patients using validated instruments, specifically the International Index of Erectile Function (IIEF) for men and the Female Sexual Function Index (FSFI) for women (52). Integrating these standardized questionnaires into clinical practice not only facilitates early detection but also legitimizes sexual health as an essential component of holistic patient care (53).

Following a positive screening result, a comprehensive and nonjudgmental sexual history should be obtained. This evaluation should characterize the onset, nature, and contextual factors of symptoms across all phases of the sexual response cycle, including desire, arousal, orgasm, and pain (54). Concurrent assessment of relationship satisfaction, psychological status, and specific concerns regarding viral transmission or fertility is equally important (55). This holistic approach helps differentiate biological, psychological, and relational contributors to SD, thereby informing subsequent management strategies.

A targeted physical examination may reveal signs of hypogonadism, such as reduced body hair or gynecomastia in male patients, or evidence of vaginal atrophy in female patients (56, 57). Essential laboratory investigations should include assessment of total and free testosterone, estradiol, luteinizing hormone (LH), follicle-stimulating hormone (FSH), and prolactin (58). These parameters help evaluate endocrine function and correlate sexual dysfunction with underlying disease activity. In select cases, such as men with erectile dysfunction refractory to initial management, specialized testing including penile Doppler ultrasound may be warranted to assess vascular integrity (59).

### 5.2 Step 2: addressing biological and pathophysiological factors

The initial therapeutic focus should center on optimizing management of the underlying liver disease and associated comorbidities (60). Ensuring patients receive a suppressive and well-tolerated NA regimen is paramount (61). For individuals experiencing significant SD, the neutral sexual side effect profile of NAs renders them preferable to interferon-based regimens. Effective virological suppression forms the foundational intervention, as it mitigates systemic inflammation and may improve overall wellbeing (62).

Concurrent management of comorbidities including diabetes, hypertension, dyslipidemia, and obesity is critical. Aggressive control of these conditions improves endothelial function and cardiometabolic health, with direct beneficial effects on sexual performance (63). A multidisciplinary approach involving primary care physicians or endocrinologists is often required to achieve optimal control.

For specific sexual symptoms, targeted pharmacotherapy provides effective intervention. In men with erectile dysfunction, phosphodiesterase 5 inhibitors such as sildenafil and tadalafil represent first line therapy. Direct evidence for CHB patients with ED is limited to a small sample study; however, substantial data from patients with metabolic syndrome and post-liver transplant patients support the efficacy of PDE5. Therefore, it can also be tried in CHB patients with ED. These agents counteract the endothelial dysfunction characteristic of CHB by enhancing nitric oxide mediated vasodilation (64). Notably, PDE5 inhibitors are mainly metabolized through hepatic CYP3A4 pathways and impaired hepatic function may increase drug exposure (65). In compensated cirrhosis, typically Child-Pugh A, treatment should be initiated at the lowest available dose and titrated carefully, whereas use should generally be avoided in Child-Pugh C, decompensated cirrhosis, or hemodynamically unstable patients (65, 66). Concomitant medications should be reviewed before prescribing. Nitrates are absolutely contraindicated, and strong CYP3A4 inhibitors, such as protease inhibitors and azole antifungals, require caution because they may increase PDE5 inhibitor exposure (65, 67). Therefore, PDE5 inhibitors may be considered in selected CHB patients with ED after individualized safety assessment. For patients with advanced liver disease or complex comorbidities, hepatology-urology coordination is advisable. For women experiencing dyspareunia, non-hormonal lubricants and vaginal moisturizers serve as initial treatment. In postmenopausal women or those with significant vaginal atrophy, low dose topical estrogen therapy initiated in consultation with a gynecologist can be highly effective. For men with confirmed symptomatic hypogonadism, testosterone replacement may improve libido and erectile function, though it requires careful risk benefit assessment including evaluation of prostate health under endocrinologist supervision (68). No CHB-specific trial exists; recommendations are extrapolated from hypogonadism guidelines for chronic illness and should be made under endocrinologist supervision.

### 5.3 Step 3: integrating psychological support and lifestyle modification

Biological interventions alone yield limited success unless paired with dedicated attention to the psychological and lifestyle determinants of SD. Patient education and counseling constitute foundational therapeutic measures. Openly discussing the high prevalence of SD among individuals with CHB helps diminish feelings of shame and isolation (69). Providing clear, accurate information regarding viral transmission risks is equally vital to dispel unfounded fears that undermine intimate relationships.

Patients experiencing clinically relevant anxiety, depression, or relational distress should be referred to mental health specialists, such as psychologists or sex therapists (70). Cognitive behavioral therapy (CBT) has demonstrated particular efficacy in modifying maladaptive thought patterns and behaviors associated with sexual activity (71). For couples facing relationship strain, couples counseling can facilitate improved communication and emotional intimacy. While not specifically trialed in CHB, strong evidence from chronic disease and sex therapy literature supports these interventions; we present them as reasonable adjuncts.

A proactive approach to lifestyle modification is equally critical. Patients benefit from structured guidance regarding the value of regular moderate intensity exercise, a balanced Mediterranean dietary pattern, sufficient sleep, and smoking cessation (72). These adjustments promote cardiovascular fitness, increase energy reserves, and stabilize mood, with each contributing to a more robust foundation for sexual health. Presenting these lifestyle changes as integral components of the treatment plan, analogous to medical prescriptions, can strengthen patient engagement and long term adherence (73). While randomized trials of exercise or Mediterranean diet on sexual function in CHB are lacking, strong evidence from cardiometabolic and chronic disease populations supports these recommendations; we therefore present them as reasonable extrapolations.

## 6 Future directions and conclusion

### 6.1 Research priorities

Advancing SD management in CHB requires focused research to address key gaps. First, mechanistic studies should clarify molecular pathways linking HBV proteins, chronic inflammation, and HPG axis dysregulation. The gut-liver-brain axis is another promising direction. Longitudinal studies should integrate biological markers (sex hormones, inflammatory profiles, endothelial function, vascular assessments) with validated measures of depression, anxiety, and HBV-related stigma to disentangle biological and psychosocial contributions. Second, cross-cultural comparative studies across regions and ethnic populations are needed to understand how stigma, sexual taboos, and patient-clinician communication patterns influence SD prevalence, reporting, and experience in CHB. This will enable culturally sensitive screening and management. Third, well-designed interventional trials (e.g., RCTs) should evaluate integrated care combining pharmacotherapy, psychological counseling, and lifestyle modification against standard care. Primary endpoints should be SD specific (validated questionnaire score changes). Secondary endpoints: antiviral adherence, health-related QoL (SF-36 or CLDQ-HBV), and anxiety/depression scores (HADS-D). Fourth, clinical trials of novel antivirals must include validated PROMs for sexual health as standard secondary endpoints to characterize side effect profiles and align with patient-centered care. Finally, tailored strategies are needed for underrepresented populations: older adults patients (polypharmacy, age-related hypogonadism), pregnant/postpartum women, and adolescents transitioning to adult care. Population-specific studies are essential for equitable management.

### 6.2 Conclusion

SD is a central determinant of quality of life for individuals with CHB, driven by biological, psychological, and social factors. The current clinical paradigm marginalizes this issue and fails to address its multifaceted nature. This review calls for an urgent shift to a holistic, biopsychosocial model of management. An integrated care pathway-including routine screening, multidisciplinary intervention, and continuous monitoring-offers a practical framework to bridge this gap. Beyond individual encounters, future research must adopt a cross-cultural perspective to examine how societal norms, stigma, and communication styles shape SD experience and management across global populations. Such knowledge is essential for developing culturally competent, equitable, and effective strategies. Embracing this comprehensive approach is not merely an enhancement of care but an ethical necessity-fundamental to achieving health equity, ensuring that all individuals with CHB, regardless of cultural or geographic context, attain not only virological suppression but also sustained sexual wellbeing.
